# Epistemic Injustice in Brain Studies of (Trans)Gender Identity

**DOI:** 10.3389/fsoc.2021.608328

**Published:** 2021-03-26

**Authors:** Eric Llaveria Caselles

**Affiliations:** Center for Interdisciplinary Women's and Gender Studies, Technical University Berlin, Berlin, Germany

**Keywords:** transgender, neuroscience, epistemology, transdisciplinarity, gender identity, trans studies

## Abstract

This study undertakes an analysis of the conceptualization of gender identity in neuroscientific studies of (trans)gender identity that contrast the brains of cisgender and transgender participants. The analysis focuses on instances of epistemic injustice that combine scientific deficiencies and the exclusion of relevant bodies of knowledge. The results of a content analysis show how the ignoring of biosocial, developmental, mosaicist, contextualist, and depathologizing approaches leads to internal conceptual inconsistencies, hermeneutical deficiencies and the upholding of questionable paradigms in the research field. Interviews with researchers involved in these brain studies reveal targeted and diffuse forms of testimonial injustice against alternative approaches, promoted by the hierarchical arrangements of research teams in combination with the careerist and economic logic of research. The analysis points to the exclusion of critical epistemologies of science and the historical oppression of trans people as epistemic agents as the underlying hermeneutical deficiencies.

## Introduction

The idea of the existence of neurological traits specific to trans people, is a culturally powerful narrative that has the potential to impact social perceptions, as well as legislative and medical regulations of trans people. Crucially, scholars from Trans and Gender Studies have elaborated a critique of the biomedical construction of trans identities and highlighted the historical and contextual heterogeneity of trans embodiments, focusing on how stigmatizing ideologies and various forms of inequality materialize into living conditions and experiences detrimental to trans people's lives (Valentine, 2007; Spade, 2010; Snorton, 2017; De Silva, 2018; Fütty, 2019). Given the relevance of these insights to any research on or with trans people, a transformative dialogue between the neuroscientists researching the brains of trans people and the knowledge being produced within Gender and Trans studies is a necessary transdisciplinary project.

In a critical analysis from a Gender Studies perspective of a study comparing the structural connectivity networks of trans and cis participants (Caselles, 2018) it became apparent that certain lines of transdisciplinary engagement were already in place. Three fundamental contributions from these engagements could build the basis for a dialogue between Gender Studies and Neuroscience on trans research^1^.

The first contribution is the challenging of the “hardwiring” paradigm still dominating neuroscientific research on sex differences. It upholds that the effect of prenatal hormones on the brain of the fetus determines its future gendered behavior, sexual orientation and gender identity. In her thorough analysis of the “hardwiring paradigm,” Rebecca Jordan-Young problematizes the systematic neglect “of the well-established evidence that the brain and the neuroendocrine system (not to mention the rest of the body) are not stable foundations from which behavior and cognition emerge, but develop and change in a constant dialectic with social and material “inputs,” including the individual's own behavior, learning, and mood states” (Jordan-Young, 2010, p. 237). A first programmatic attempt at developing a biosocial theoretical framework of the emergence of sex-related differences has been undertaken by Fausto-Sterling et al. (2011a,b). Wood and Eagly (2009, 2012) have also proposed a biosocial concept of gender identity and gender role socialization. Central to this biosocial and developmental approach is the acknowledgment of brain plasticity, meaning that “the brain changes both structurally and functionally in response to the environment and experience” and that “an intrinsic feature of the brain is its sociocultural context dependence” (Han et al., 2013, p. 338, see also Kolb and Gibb, 2014).

The second contribution is the concept of brain mosaicism which challenges the notion of brain sexual dimorphism. The dimorphism model stems from the 3G-model of sex, which groups the variance of genetic, gonadal and genital expressions in a male and a female group^2^. Daphna Joel and her team argue that thinking of the brain as dimorphic, that is, as existing in a male or female variation, is a misrepresentation. In a review of more than 1,400 human brains, Joel and her team found that sex/gender differences in the human brain are neither highly dimorphic, nor internally consistent, “even when considering only the small group of brain features that show the largest sex/gender differences, each brain is a unique mosaic of features” (Joel et al., 2015, p. 15472). The same team have also empirically questioned the idea that core gender identity is clearly binary in cis population (Joel et al., 2014). They developed a Multi-Gender Identity Questionnaire, administered it to cis and trans participants and found “that the current view of gender identity as binary and unitary does not reflect the gender experience of many “normative” individuals” (Joel et al., 2014, p. 315).

Finally, the third contribution stems from the changes in the diagnostic categories and criteria for trans people. In the DSM-5 [APA (American Psychiatric Association), 2013], the diagnosis changed from “gender identity disorder” to “gender dysphoria.” The aim of the change was to depathologize gender identity and to focus instead on the suffering or discomfort of trans and gender diverse people. The terminology used in the DSM-5 describes gender identity and gender roles as spectrums and avoids binary and dichotomous logic, thereby acknowledging the existence of gender identity variance. Furthermore, the term “gender” has been used instead of “sex.” Sexual orientation, which was a specifier in the DSM-IV, has also been removed (Cohen-Kettenis and Pfäfflin, 2010; Beek et al., 2016). But what makes the DSM-5 so radically different from the previous editions is the shift in its understanding of medical authority. The DSM-5 experts discussed the diagnostic criteria explicitly in relation to the stigmatization of trans people, as well as in relation to access to healthcare. They challenge that the notion that science can define what is regarded as normal or pathological: “There are no scientifically based criteria to differentiate normal and pathological gender identity, and the manner in which any gender identity develops remains unknown and a matter of theoretical speculation” (Drescher et al., 2012, p. 573).

In the aforementioned analysis of a study of the connectivity networks of cis and trans participants (Caselles, 2018) it became clear that these contributions were not being discussed - despite their direct relevance. Instead, the neuroscientists remained committed to the hardwiring paradigm, binary models of sex/gender and pathologizing understandings of trans identity. Was this exclusion specific to this paper or did it affect the whole field? Did the exclusion result from ignorance or was it intentional?

Normative theories of epistemic injustice provide a framework to think about these questions. Its central idea is that discrimination of people as epistemic agents leads not only to the disadvantage of members of groups discriminated against, but to an impoverishment of knowledge overall: “knowledge that is passed on to a hearer is not received. This is an epistemic disadvantage to the individual hearer, and a moment of dysfunction in the overall epistemic practice or system.” (Fricker, 2007, p. 43).

José Medina and Miranda Fricker, two of the main theorists of epistemic injustice, distinguish between testimonial injustice and hermeneutical injustice. The core of testimonial injustice is a prejudicial dysfunction in the attribution of credibility by a hearer. Credibility excess and credibility deficit are forms of testimonial injustice, which are systematically linked to each other. Both Medina and Fricker qualify as unjust those forms of credibility excess or deficit that are systematic, that is, “that track the subject through different dimension of social activity - economic, educational, professional, sexual, legal, political, religious, and so on” (Fricker, 2007, p. 27). Other forms of testimonial injustice are pre-emptive testimonial injustice, in which a group is excluded from participating in epistemic exchange, and epistemic objectification, that is, the denial of epistemic subjectivity to certain groups by confining them to passivity and excluding them from epistemic co-operative exchange.

The basis for hermeneutical injustice is the fact that individuals' knowledge of the social world and of themselves is interpretative, meaning that we are all dependent on a pool of hermeneutical resources to make sense of our social experiences. Injustice occurs when collectively available resources to understand oneself and one's social experiences are unevenly informed by the experiences of some social groups to the exclusion of others. Hermeneutical injustice is “the injustice of having some significant area of one's social experience obscured from collective understanding owing to a structural identity prejudice in the collective hermeneutical resource” (Fricker, 2007, p. 155).

My aim is to apply the theoretical framework of epistemic injustice theories to empirically analyze an actual epistemic situation between neuroscientific studies of (trans)gender identity and alternative approaches. In order to do this, I combine epistemic injustice theories with an ethnomethodological approach to the study of scientific practices, which reveals the centrality of testimonial and hermeneutical dimensions of scientific facts. Latour has argued that it is not the inherent properties of statements that make them true, but the incorporation of these statements into new ones by other actors. In this sense, facts are collective accomplishments with an essential communicative dimension: “You may have written a paper that settles a fierce controversy once and for all, but if readers ignore it cannot be turned into a fact.” (Latour, 1987, p. 40).

Hermeneutical practices in the production of scientific knowledge occur at two levels. The first one is based on the interpretation of observations. At this level, the systematic privileging of certain interpretations can constitute a form of hermeneutic marginalization. The second level is connected to the understanding and meaning of science itself. Differences are found for example between monist and pluralist understandings of science (Kellert et al., 2006, p. xi).

Using theories of epistemic injustice involves a commitment toward a more just production of knowledge. It is in this sense a necessarily interventionist project. Based on a vision of dissent as a democratic epistemic practice, Medina advocates for epistemic friction, which he defines as “contending with,” rather than “contending against” (Medina, 2013, p. 16). In this account of epistemic cooperation, he defends the principles of acknowledgment, engagement, and epistemic equilibrium. The first of which means that “all forces that we encounter must be acknowledged and, insofar as it becomes possible, they must be in some way engaged,” the second one is the imperative to search “for equilibrium in the interplay of cognitive forces, without some forces overpowering others, without some cognitive influences becoming unchecked and unbalanced” (Medina, 2013, p. 50). He places a special hermeneutical responsibility on institutions and people in positions of power, but stresses that “we all share the collective responsibility to facilitate the hermeneutical agency of all communicators, especially if they have been marginalized” (Medina, 2013, p. 110).

This political and ethical commitment also is found within feminist philosophy of science. Donna Haraway's concept of situated knowledges captures this sense of the individual and collective responsibility of researchers within an understanding of science as historically contingent, constituted through language and meaning, as well as committed to “faithful accounts of a “real” world” (Haraway, 1988, p. 579). Upholding the value of embodied objectivity against traditional epistemology and social constructivist relativism, Haraway argues for “partial, locatable, critical knowledges sustaining the possibility of webs of connection called solidarity in politics and shared conversations in epistemology” (Haraway, 1988, p. 584). The possibility of objectivity and rational knowledge lies then in the “process of ongoing critical interpretation among “fields” of interpreters and decoders,” in knowledges “ruled by partial sight and limited voice (.) for the sake of the connections and unexpected openings (.)” (Haraway, 1988, p. 587). I see this account as in harmony with Medina's normative account of epistemic cooperation that provides the theoretical and normative basis for my research.

Building on these frameworks I formulate my research question as follows:

Which forms of epistemic injustice can be identified in the conceptualization of gender identity in the brain studies of (trans)gender identity?^3^

Within the context of this research question, I align with an approach that can be defined as biosocial, developmental, mosaicist, contextualist, and depathologizing. My understanding of “conceptualization” includes both the formal definitions presented in the published studies and the process by which these definitions were established.

In order to empirically assess epistemic injustice, a transparent operationalization is needed. The criteria formulated below are tailored to the context of scientific research and target the moments of decision-making among alternative options within the research process, including the formulation of research questions, design of experiments or the interpretation of findings. The criteria are not meant to lead to conclusive “yes/no” answers on the question of whether epistemic injustice is to be found in a particular case. Instead, they are meant to provide an evaluative framework to interpret the data gathered.

First, the question of epistemic injustice can only be adequately raised if a number of preconditions are met in an epistemic situation:

A1. Multiple epistemic agents (individual, collective, institutional) participate actively in the production of knowledge^4^.
A2. There is a shared question or inquiry involving all epistemic agents.
A3. The knowledge produced by the epistemic agents follows a shared set of values and rules, and is of relevance to the inquiry.
A4. There must be a power differential in the epistemic situation that corresponds to relations of oppression active in society.

The epistemic situation of this study involves on the one hand the scholars advancing biosocial, developmental, mosaicist, contextualized and depathologizing approaches to brain research and sex/gender and trans identities. On the other hand, it involves brain studies of gender identity (BSGI). The term “brain studies of gender identity” is used in this paper in a narrow sense. It refers to neuroimaging studies that state “gender identity” as their main object of research, operate by comparing the brain structure and function of trans and cis participants, and aim toward a neurological theory of gender identity formation. Consistent with my praxeological approach, the use of the term is descriptive and captures how the studies present themselves. It is important to note that this use of terminology reproduces a problematic and undifferentiated notion of gender identity as a one-dimensional self-contained category, a conceptual issue that is investigated and discussed in sections Conceptualization of Gender Identity in Published Brain Studies of (Trans)Gender Identity, Epistemic Attitudes From Researchers of Brain Studies of (Trans)Gender Identity, and On Epistemic Injustice in Brain Studies of (Trans)Gender Identity of this paper. The BSGI do not include neuroimaging studies with trans participants looking into the effects of hormone replacement therapy on brain structure or function (see for example Burke et al., 2018), nor studies with trans participants with research objects that are not explicitly gender identity, such as ostracism (for example Mueller et al., 2018) or reaction to stimulation of body parts (see for example Case et al., 2017).

These two sets of epistemic agents configure an epistemic situation in which they are all directly involved in seeking to understand gender identity and are accountable to scientific standards for empirical research, even if they deploy different methodologies. As a baseline, all of the epistemic agents hold positions in universities or research institutions and have published their work in peer review journals to which the other researchers have access.

The power differential between these epistemic agents (A4) is, however, more difficult to argue. This is because I have narrowed down the epistemic injustice situation as occurring within the realm of science, that is, between epistemic agents that qualify as scientists, which is a position of social privilege. The difference between the epistemic agents that I am considering is not one of gender identity, sexual orientation, race, class or other category of social inequality. There is however a key difference in that some epistemic agents operate within dominant and hegemonic discourses, while biosocial, developmental, mosaicist, contextualist, and depathologizing views represent counter-hegemonic positions. The counter-hegemonic stance is directed against two central dispositives of western modernity: the sex/gender binary norm, and the scientific authority over what constitutes nature. Historically, these two strands have come together in the normative legal and biomedical definitions of manhood and womanhood as the only two possibilities of social and political existence in western nation-states.

Thus, the four preconditions that enable one to analyze whether an epistemic situation is shaped by epistemic unjust behavior are met. The focus of my inquiry are therefore the following four conditions, which establish the framework to assess epistemic injustice and guide my research design:

B1. The wrong of the dominant epistemic agent must amount to blocking the epistemic labor of others, devaluating the epistemic labor of others, and/or appropriating the epistemic labor of others.
B2. There must be a form of exclusion or limitation in the participation in the production of knowledge that keeps epistemic agents isolated from one another and/or there must be a breach in the relationship of trust between the epistemic agents involved.
B3. The harming of the oppressed epistemic agent must benefit the dominant epistemic agent in the perpetuation of the privileges granted through the relations of dominance and oppression which structure society.
B4. The harm produced by the dominant epistemic agent must amount to a failing within the rules of the epistemic system, to a failure of the epistemic system or to the inadequacy of the system altogether.

In order to make the criteria less exigent, I narrow them to alternatives that operate within the same epistemic system, in this case, empirical scientific research in general and biology and neuroscience in particular. This is to be seen as a strategic restriction, since I would defend that epistemic agents have a responsibility to pay attention and engage with knowledge from epistemic systems other than their own.

## Materials and Methods

### Content Analysis

The first part of the study is a qualitative content analysis (Mayring, 2015; Krippendorff, 2018) of BSGI to analyze how gender identity is defined in published studies. The qualitative content analysis can establish whether the definitions of gender identity meet quality criteria of conceptual work such as clarity, specificity, coherence, or consistency. This analysis can also show whether the conceptualization engages with biosocial, developmental, mosaicist, contextualist, and depathologizing approaches. Therefore, the qualitative content analysis is an adequate method to evaluate the conditions B1 (blocking, devaluing, or appropriating the epistemic labor of others), B2 (exclusion or limitation in the participation in the production of knowledge), and B4 (failing within the values of the epistemic system).

The content analysis was divided into two steps: first, a detailed analysis of four early studies (2011–2014), and second, a targeted analysis of six recent studies (2016–2018). The sample for the first analysis was based on the relevance of the findings for the BSGI field: Savic and Arver (2011), Rametti et al. (2011a,b), and Kranz et al. (2014). The sample of the recent studies was based on publication date, inclusion of different approaches, relevance of the findings, and availability of researchers for the interview: Guillamón et al. (2016), Burke et al. (2017), Feusner et al. (2017), Manzouri et al. (2017), Nota et al. (2017), and Manzouri and Savic (2018).

The procedure for the analysis was developed with a pre-test of a previous study (Berglund et al., 2008). In the analysis I considered the terms “gender identity,” “sex,” “gender,” “transgender” (and related terms such as “gender dysphoria,” “gender identity disorder,” “transsexualism”), “women/female,” “men/masculine” and terms related to “sexual orientation.” I accounted for explicit definitions and the use of the terms^5^.

In the analysis of the early studies, I conducted a quantitative assessment of the frequency of use of the terms^6^. For the qualitative analysis of the most frequently used terms, I took into account explicit definition and uses of the terms. I differentiated the uses within theoretical expositions, operationalizations or interpretations of findings. I also analyzed the use of different terms in relation to each other. Then, I analyzed expressions of sexual differentiation of the brain, that is, expressions that communicate the measurements obtained in a study (own or other) and interpret them in relation to O/A hypotheses. Finally, I included in the analysis explicit definition of the considered terms from cited theoretical papers. The findings of the analysis of the early studies are presented in section Conceptualization of Gender Identity in the Recent Brain Studies of (Trans)Gender Identity (2011–2014).

The analysis of the recent studies was a comparative analysis, focused on changes in relation to the earlier studies. In order to do this, I accounted for theoretical shifts in the field. The conceptualizations of gender identity were then analyzed separately for the two hypotheses proposed in the recent studies. I analyzed explicit definitions and uses of “gender identity” and the aforementioned terms in the theoretical expositions, operationalization, and interpretation of findings. The findings of the analysis of the recent studies are presented in section Conceptualization of Gender Identity in the Recent Brain Studies of (Trans)Gender Identity (2016–2018), while a joint discussion of the findings in the early and recent brain studies follows in section Summary of findings of the conceptualization of gender identity in published brain studies of (trans)gender identity.

### Expert Interviews

The second part of the study is an assessment of the researchers' epistemic attitudes toward the alternative approaches. I used qualitative expert interviews for this purpose (Kaiser, 2014). The interviews combine exploratory questions to generate new insights in the conceptualization process, with structured questions to evaluate the following necessary conditions for epistemic injustice: B1 (blocking, devaluing or appropriating the epistemic labor of others), B2 (exclusion or limitation in the participation in the production of knowledge), B3 (perpetuation of privilege granted through relations of dominance and oppression), and B4 (failing within the values of the epistemic system, failure or inadequacy of the epistemic system altogether).

The interview script was designed to introduce epistemic friction by asking about conceptual problems of the BSGI, as well as about biosocial, developmental, mosaicist, contextualist, and depathologizing approaches. The challenge was to avoid an oppositional framing and instead promote a dialogue between dissenting stances.

I approached this by establishing a common ground between myself as the interviewer, the BSGI researchers, and alternative conceptualizations. This common ground was based on four openings in the recent studies: (1) the dismissal of the inverted brains hypothesis in favor of a reconceptualization of trans brains as a composite of masculinized and feminized traits, potentially opening the research toward a mosaicist model of brain sex differentiation, (2) emphasis on development which enables the discussion of brain plasticity and environmental factors, potentially moving away from biological determinist models of gender identity, (3) the introduction the diagnosis of “gender dysphoria” in the DSM-5 which acknowledges non-binary gender identities, potentially opening the field toward multidimensional and socially contextualized understanding of gender identity for trans people, and toward a denaturalization of diagnostic categories, and (4) consideration of social experience as a factor shaping brain networks, potentially opening research toward multidimensional, intersectional and socially contextualized understandings of gender identity for both trans and cis people.

I developed questions that create a space for discussion: “How relevant do you consider theory x for the neuroscientific study of gender identity?,” “What benefits and problems do you see in approach x at a theoretical and methodological level?,” “Was this conceptualization x topic of discussion?.” For questions with an either-or logic, I attempted to establish in the formulation a collaborative focus, for example: “Do you think that x should inform neuroscientific research on gender identity?^7^.”

I sent interview requests to 11 researchers and received 4 positive responses, 4 declines, and 3 unanswered. I conducted four interviews that lasted between 37 and 70 min and recorded the audio. I adapted the interview script to each researcher focusing on the area of expertise. I transcribed the interviews following a simple transcription method (Dressing and Pehl, 2015). A first transcription was sent to the interviewees for revision. In order to create a relationship of trust, but also to reflect potential changes in the epistemic attitude of the interviewees, I allowed them to introduce modifications to the transcription. One researcher decided to retract the interview after reading the transcript, which is why the analysis is limited to three interviews. Researcher B introduced modifications in the transcript, which became much shorter and closer to a written text and left out many questions and answers. The interview transcripts were then anonymized.

The interviews were analyzed following a simple qualitative content analysis focusing on two categories. The first, communicative context, reflects the interviewees' construction of the social field^8^ in which they situate their own work and that provides the background for their conceptual decision-making. This category enables a description of the epistemic attitude in relation to the constraints that dominate the social field. The findings of this analysis are presented in section Communicative Contexts. The second category is epistemic behavior, specifically in relation to the conceptual problems and alternative approaches. This category is a praxeological one, framing the reaction in terms of “doing”: what does the researcher do with the conflict? How does the researcher handle it? This takes into account both the content level and the performative level of communication, paying attention to the arguments offered and how they function within a range from refusal or blocking to engagement and agreement. The results of this analysis are presented in section Epistemic Behavior.

## Conceptualization of Gender identity in Published Brain Studies of (Trans)Gender Identity

### Conceptualization of Gender Identity in the Recent Brain Studies of (Trans)Gender Identity (2011–2014)

#### Frequency of Use of Sex/Gender Related Terms

The use of sex/gender related terms differs in absolute numbers between the three studies, but shows a consistent pattern (see Table 1). The terms most used are “men/male” and “women/female,” followed by terms related to “trans” and “sex.” The numbers show a consistently low frequency of use of “gender” related terms, despite it being the main object of the studies' research.

**Table 1:** Absolute and relative frequency of use of sex/gender related terms in selected studies.

	**Savic and Arver (****2011****)**		**Rametti et al. (****2011a****)**		**Kranz et al. (****2014****)**		**Total**	
“sex”	50	25,6%	44	19,4%	54	18,1%	148	20,55%
“gender”	2	1%	18	7,9%	27	9%	47	6,5%
“men/male,” “women/female”	59	30,3%	82	36,1%	110	36,9%	251	34,9%
“sexuality”	40	20,5%	12	5,3%	24	8,1%	76	10,55%
“trans”	44	22,6%	71	31,3%	83	27,9%	198	27,5%
	195	100%	227	100%	298	100%	720	100%

#### Explicit Definitions of Gender Identity

None of the four studies included an explicit definition of “gender identity.” The most elaborate were formulations such as “perceptions of the own sex” (Savic and Arver, 2011) or “the male controls have a gender identity as men (…) and control women have a gender identity as women” (Rametti et al., 2011b). This is remarkably poor considering the centrality of gender identity in the research question.

The theoretical papers cited in the studies contained two brief definitions, both within a parenthesis. In the first one, gender identity is distinguished from “sex,” defined as a form of belonging, and limited to a masculine or feminine identity as mutually exclusive and homogeneous categories: “Gender identity (gender identity refers to an identity experience expressed in terms of masculine or feminine “belongingness,” independent of the anatomical reality of the sex) (.)” (Swaab, 2004, p. 303). In the second one, this “feeling of belonging” is related to gender, without explaining the term any further and remaining within the male-female dichotomy: “… gender identity (the conviction that one belongs to the male or female gender).” (Bao and Swaab, 2011, p. 215). Both definitions are embedded in explanations of transsexuality, establishing a normative dimension by which the main aspect of gender identity is the distinction between ordered (cis) or disordered (trans). This subordinates the concept of gender identity to the definition of transsexuality and centers cis identities as an invisible norm.

#### Etiological Definition of Gender Identity

The theoretical framework of the four studies is the brain organization/activation (O/A) hypothesis, particularly the discussion thereof by Swaab (2004, 2007), Garcia-Falgueras and Swaab (2008), and Bao and Swaab (2011). The O/A hypothesis proposes that a permanent structuring (“hardwiring”) of the brain into a male or a female variation occurs based on the influence of gonadal testosterone on the developing brain of the fetus and immediately after birth. These “hardwired” brain patters are proposed to cause differences between men and women in gender and gender identity, including behavior, personality traits and feeling of belonging (see Bao and Swaab, 2011, p. 215).

Gender identity is introduced in the hypothesis to explain transsexuality through the separate timing of genital and brain differentiation during pregnancy. The separate timing opens up the possibility of changes in the hormonal environment in which brain and genital differentiation happen:

“These fetal and neonatal peaks of testosterone, together with functional changes in steroid receptors, are thought to program to a major degree the development of structures and circuits in a boy's brain for the rest of his life. As sexual differentiation of the genitals takes places much earlier in development (i.e., in the first 2 months of pregnancy) than sexual differentiation of the brain (the second half of pregnancy), these two processes may be influenced independently. In rare cases, this may result in transsexuality, i.e., people with male sex organs who nevertheless have a female identity, or vice versa. It also means that in the event of an ambiguous sex organ at birth, the degree of masculinization of the genitals may not always reflect the degree of masculinization of the brain” (Bao and Swaab, 2011, p. 215).

The implication of this etiological hypothesis is that chromosomal and genital sex need to be seen in all people as independent from gender identity and, by extension, that neither chromosomal xx/xy variation, nor the presence of a penis or a vagina, can be used as reliable indicators of the hormonal environment during the fetal brain development. This implication is ignored by the authors in BSGI and the proponents of the O/A hypothesis, but it has far reaching consequences for the whole field of studies of sex/gender differences in the brain. Studies that use chromosomal, genital or gonadal sex as indicators of the hormonal environment during fetal brain development or as an indicator for the gender or gender identity of the participants can't be seen as reliable. Based on the O/A hypothesis itself, this combination of inferences is not valid, meaning that gender and gender identity need to be assessed in all people independently from 3G-sex and that 3G-sex can't be used as a reliable indicator of hormonal environment during brain development. The undetected logical inconsistency in the application of the hypothesis in BSGI shows the detrimental impact of unreflected cultural and normative assumptions of sex/gender on the research field.

#### Operational Definitions of Gender Identity

In all four studies, participants were selected based on sex and gender identity. None of the studies mentions how the gender identity of the cis participants was established. At the same time, trans participants underwent an exhaustive control of their gender identity based on diagnostic procedures and criteria of the DSM-IV and ICD-10. The fact that only the gender identity of trans participants was operationalized shows the extent to which the conceptualization of gender identity is dependent of the ordered/disordered dimension of a medical diagnosis and how a cis bias stands in the way of a thorough interrogation of the category of gender identity.

The use of a medical category for the assessment of gender identity in combination with the etiological model proposed by the O/A hypothesis by which gender identity is hardwired through the effect of prenatal hormones on the brain leads to a fundamental hermeneutical problem in the BSGI. The biologization of transsexuality erases the historical and political dimension not only of sex and gender, but also of medical categories and techniques.

#### On the Use of “Brain Sex,” “Biological Sex”

In the studies, the term sex is used in expressions such as “biological sex” and “brain sex.” In “biological sex” it refers to a series of elements such as genital phenotype, reproductive organs, gonads, production of androgens and estrogens, and chromosomes. These factors are linked by a chain of events and processes. Within the O/A hypothesis, the development of the brain is determined by “sex” in the sense that it is assumed to be shaped permanently by the gonadal hormones. It is in this sense that “brain sex” can be understood as expressing the causal subordination of brain structure and function, as well as their outcomes (behavior, attitudes, cognition, emotion, identity, etc.), to factors of biological sex (see Savic et al., 2010, p. 15).

The term “brain sex” is misleading in a crucial way. Taking the O/A hypothesis seriously, that genitals and brain differentiation occur at different times during pregnancy, “sex” in “brain sex” stands for the hormonal environment during the brain development phase of the fetus in which the “gender” gets hardwired. However, as the hypothesis proposes in the explanation of transsexuality, this hormonal environment can't be assumed from chromosomal, genital or gonadal sex, and the outcome as male/female gender identity can't be predicted by either gonadal sex or chromosomal sex or genital sex. Thus, the use of “brain sex” creates a false correspondence between chromosomal sex, gonadal sex, genitals, brain structure, gender, and gender identity in a male expression or a female expression.

#### Definition of Transgender Identities and Sexual Orientation

The most elaborate definition in the studies refers to terms related to transgender identities. The authors of all studies include a diagnostic definition based on the criteria of DSM-IV and ICD-10:

“(1) A desire to live and be accepted as a member of the opposite sex, usually accompanied by a sense of discomfort with the subject's anatomical sex and a wish to have surgery and hormonal treatment to make the body as congruent as possible with the body of the preferred sex.
(2) The transsexual identity has existed for at least 2 years.
(3) The syndrome cannot be explained by any other psychiatric disorder or by chromosomal abnormality. Thus, any evidence of an abnormal male phenotype or genotype (i.e., hypospadias, cryptorchism, micropenis, and chromosome complement other than 46XY) excluded enrollment to the study” (Savic and Arver, 2011, p. 2,526).

Based on the O/A hypothesis, transgender identity is also understood as “a mismatch between gender-specific brain development and the development of body and genitals” (Kranz et al., 2014). They combine these two definitions with the typological differentiation of trans people in a “homosexual” and “non-homosexual” category based on Blanchard's discredited hypothesis. The typological definition is used by the researchers to control for sexual orientation as a factor.

“All FtM transsexuals selected had early-onset gender non-conformity (before puberty), were erotically attracted to females, and wanted sex reassignment (Gómez-Gil et al., 2009). This group corresponds to the one typically referred to as “homosexual type” (Blanchard et al., 1987; Smith et al., 2005; but see Gooren, 2006). Sexual orientation in patients was established by asking what partner (a man, a woman, both or neither) the patient would prefer or feel attraction to if they were completely free to choose and the body did not interfere” (Rametti et al., 2011b, p. 950).

The reason for the assessment of sexual orientation is that the O/A hypothesis also applies to the “hardwiring” of a sexual orientation. Therefore, the variable sexual orientation is assessed to either limit the selection of participants to “heterosexuality” or to use it as covariate. Following Gooren (2006), Moser (2010) and Veale et al. (2012) critique of the Blanchard typology, the operationalization of sexual orientation for the cis and trans participants is contradictory. For example, trans men attracted to women are defined as homosexual (Rametti et al., 2011a, p. 200). The Kranz et al. study is the only one that accounts for different possibilities to operationalize sexual orientation based on a scale of attraction toward males and females, on a spectrum of homosexuality and heterosexuality based on genetic sex and on the same spectrum based on gender identity (see Kranz et al., 2014, p. 15469).

The studies' use of multiple definitions of transgender identities without acknowledging incompatibilities and the contradictory assessment of sexual orientation contribute to the lack of conceptual clarity and accountability.

### Conceptualization of Gender Identity in the Recent Brain Studies of (Trans)Gender Identity (2016–2018)

In order to assess the recent BSGI it is necessary to account for theoretical shifts in the field. The changes were necessary because the findings didn't show a “brain sex reversal” in trans participants, but a mix of traits: “the MtF brain is not completely feminized but presents a mixture of masculine, feminine, and demasculinized traits” (Guillamón et al., 2016, p. 1627). The first hypothesis used to explain these findings is the cortical development hypothesis (CD), which adapts the O/A theory to match the findings. The second hypothesis is the self-referential thinking and body perception hypothesis (SR/BP), which operates within neurological theories of the self. Another development that affected recent studies was the release of the DSM-5, which introduced relevant changes in nomenclature from “gender identity disorder” to “gender dysphoria,” and demonstrated a deeper understanding of transgender identities.

#### Cortical Development Hypothesis

This hypothesis is presented in a review paper and has not been empirically tested. It proposes “a slowing (or a stop) in the cortical thinning process in females, MtFs, and FtMs compared to the thinning process in males,” which would create different cortical phenotypes: “this hypothetical process, based on differential developmental processes in specific cortical regions, would influence the development of gender identity for all: male, female, MtF, and FtM” (Guillamón et al., 2016, p. 1637).

In relation to the conceptualization of gender identity, the CD hypothesis does not provide any further elaboration on the O/A model. The shift is that gender identity is defined not through a male and female pattern that is reversed in the trans brain, but through a “thinner than male” cortical thickness pattern. While the first pattern was proposed based on a binary oppositional concept of sex and gender, this new pattern has no logical correspondence to the conceptual definition of sex/gender, which is maintained as binary and oppositional.

The CD hypothesis does not integrate the changes in conceptualization of the DSM-5, despite citing it as a reference. Instead, the CD hypothesis makes extensive use of Blanchard's typology of transsexuality and “feminine essence theory” (Blanchard, 2005, 2008), disregarding it's sexist and homophobic logic and its incompatibility with the understanding of gender incongruence of the DSM-5.

#### Self-Referential Thinking and Body Perception Hypothesis

This alternative hypothesis seeks to describe the networks involved in accomplishing tasks such as recognizing one's own body as one's own. It includes a definition of gender identity that considers a series of factors: “gender identity denotes a complex interrelationship among an individual's genital sex, one's internal sense of self, and one's outward presentations and behaviors (gender expression)” (Manzouri and Savic, 2018, p. 1). However, there is no acknowledgment that the internal sense of self and one's outward presentations and behaviors are related to sex/gender, due to the fact that neurological theories of the self don't have a concept of gender (see Northoff et al., 2006, p. 454). Gender dysphoria is thus redefined as “body dysphoria and body-related avoidance” (Feusner et al., 2017, p. 965), erasing gender as a dimension.

The conceptualization of gender dysphoria away from sex/gender models introduces a shift in the question of causation. The authors move away from a “neurobiological determinant” to a “neurobiological substrate,” taking into account plasticity and development in what can be understood as a shift toward a biosocial reconceptualization (see Manzouri et al., 2017, p. 1008). Some studies introduce a developmental understanding of gender dysphoria, focusing on aging and brain maturation and activational effects of hormones in puberty, and disregarding the effects of differing social experiences (see Nota et al., 2017).

In the SR/BP hypothesis sexual orientation figures as a separate phenomenon, leading to the interpretation that “the neuroanatomical signature of transgenderism is related to brain areas processing the perception of self and body ownership, whereas homosexuality seems to be associated with less cerebral sexual differentiation” (Burke et al., 2017, p. 1). The conceptual entanglement of gender identity and sexual orientation is not considered.

### Summary of Findings of the Conceptualization of Gender Identity in Published Brain Studies of (Trans)Gender Identity

Overall, the definitions and use of the terms “gender identity,” “sex,” and “gender” fail to meet sufficient levels of accuracy and differentiation. In addition to the studies' general lack of clarity, I see three severe conceptual problems.

The first conceptual problem lies in their disregard of the theoretical and methodological implications of the postulated temporal separation of 3G-sex and gender identity. This problem can be understood as a form of internal conceptual inconsistency. In relation to the conditions defined in the operationalization of epistemic injustice, this conceptual inconsistency represents a problem within the rules of the experimental method.

The second problem is the hermeneutical misconception by which cultural norms, practices and techniques of gender are naturalized and turned into biological entities. This is evident in the usage of transsexuality as the only explicit frame of reference for gender identity, as well as in the failure to operationalize gender identity for cis participants. The hermeneutical misconception points to a possible inadequacy of the theoretical and experimental approach of the BSGI to acknowledge the socio-cultural dimension of its research object and stands in the way of a complex understanding of gender identity for all people.

The third problem is the upholding of questionable paradigms, such as biological reductionism and determinism, as well as the binary model of thinking about sex/gender and brain. These frameworks contradict knowledge of the biosocial and dynamic quality of brain development and the evidence of sex/gender diversity presented in the introduction. While there is an emphasis on the developmental logic in both SR/BP and the CD hypotheses, and the SR/BP is open to the acknowledgment of brain plasticity, no social or cultural variables affecting gender identity or brain development were considered relevant. While it is important to acknowledge the opening and to take its potential for future research seriously, both hypotheses remain attached to reductionist thinking. This third conceptual problem represents a failure to acknowledge relevant bodies of work, and thus a failure of the epistemic system to detect and prevent harmful ignorance.

## Epistemic Attitudes From Researchers of Brain Studies of (Trans)Gender Identity

### Communicative Contexts

The interviewed researchers of the BSGI belong to different research teams and have different tasks, experience levels and academic status. This leads to contrasting perceptions of the work in the research teams, the broader scientific community and the socio-political context of the studies.

#### Researcher A

Researcher A works as a doctor in a gender clinic and is thus accountable to diagnostic manuals such as the ICD and DSM, national laws on name and sex change registration and healthcare system regulatory bodies. Researcher A also works closely with trans patients and trans organizations and is aware of the conflict between the regulatory framework of the gender clinic and the healthcare needs of trans people who go there. Researcher A is recruited to work on the BSGI and entered the research team in a subordinated relationship with the principal investigator, who is the funding receiver and ultimate decision-maker. The question of access to funding highlights the entanglements between career logic and the logic of knowledge production. However, researcher A points to the crucial difference of having a regular salary as a doctor and the situation of researchers, who have to “spend half of their work time to apply for money (…) and in that, they need to sell.”

Researcher A brings into the team an awareness of the political dimension of scientific research on trans topics: “(.) you have to fight if you do studies with people who are not in trans medicine, they want to use what they think is simple language. So, why complicate it, it's a female-to-male (.) and there's whole other studies using this, we cannot not use it.” For researcher A the political dimension of medical and scientific work with trans people makes it necessary for researchers to intervene in public debates to prevent harmful use of findings. This involves a self-positioning in relation to the distributions of privilege and power involved in research: “I am privileged, I have a reputation, I have a salary (…) and I can feel stressed by being in these sometimes hostile surroundings (…). But the trans patient (…) is of course even more in this needle of a hurricane.” Researcher A sees a need to involve trans people when planning research to think about “what questions are more urgent to answer, what is interesting, what is important?.” This stance of Researcher A includes an awareness that trans people “have different views of things” and that some trans organizations think that there should be “no medical people at all.”

#### Researcher B

Researcher B has been studying sex differences in the brains of mammals for over four decades. Their involvement in BSGI with trans patients was motivated by technological developments in neuroimaging techniques. Researcher B sees the collaboration with other research groups and institutions as a “functional team that is constituted to answer questions about gender identity and involves several universities and hospitals.” The gender unit is for researcher B purely instrumental: “the gender unit of the hospital is the one that has *nourished* all the studies that we have carried out” (my emphasis).

As a principal investigator, Researcher B seeks to enter into dialogue with the wider scientific community working on the same or related questions. Central to being able to participate in this dialogue is the use of a shared methodological approach that facilitates the integration of results “I decided, according to previous researchers, to approach the issue of gender identity in a very simple way: contrasting the brains of transgender men and women with non-transgender men and women.”

In researcher B's understanding, science and politics should be kept separate, since “research needs serenity and not looking for results that confirm particular ideas about what human nature looks like.” Researcher B rejects the use of terms such as “cisgender” because it was “invented by Volkmar Sigusch more than 20 years ago” and because it is not known by “people in the street.” This raises the question of what makes the category of “cisgender” more “invented” or politically motivated than categories such as “transsexualism,” “gynephilia,” or “gender dysphoria.” This shows that the boundary between natural/scientific categories and socio-political categories for researcher B is not dependent on the origin of the categories but of how established they are in scientific discourse. At the same time, Researcher B holds the view that “it is important (.) to use a vocabulary that is respectful and recognizes the variety of our species.”

#### Researcher C

Researcher C got involved in the BSGI in order to complete a PhD and was new to the topic at the time. Researcher C worked on BSGI in two different research groups. For researcher C, their participation in the research is structured around their relationship to a supervisor, who owns the research data. Researcher C also highlights the importance of funding policies and the difficulties to get money as “the transgender topic is not super sexy to funders,” mainly because “it's still only a small minority that are affected by it.” This economic situation keeps the field of BSGI small and concentrated into a few established teams. The only way for younger or less established researchers who are interested in pursuing innovative hypotheses is to work unpaid “in the evening hours and weekends.”

In the entanglement between the career logic and the logic of knowledge production there is a tension between collaboration and exchange on the one hand and protectionism on the other hand. Researcher C recounts instances in which collaboration requests from researchers with alternative approaches were denied because the heads of research were “very suspicious on opening up, allowing others to test their hypotheses on their data.” Researcher C participates and advocates for collaborative projects with shared data pools as a way to avoid the concentration of power in the knowledge production in “certain personalities” who decide “why certain hypotheses were tested and others were not.”

### Epistemic Behavior

#### Biological Determinism and Biosocial Approaches

In the first conceptual question I asked the interviewees thought how relevant they considered biosocial frameworks for the neuroscientific understanding of gender identity and gender incongruence.

Researcher A's understanding of gender identity was based on Fausto-Sterling's theory of gender identity development, rejecting mechanistic and reductionist models: “the concept of self could not be (…) in one nucleus deciding if we are male or female as a matter if they are big or small. It must be a network giving us this. And I also think that the way we see our body is also in a network - that we, no matter whether we are gender incongruent or not, but how we see our body is formed by connection between your body and your brain and how you interpret that.”

Researcher B preferred in this question to their own theory as “a first explanation of all possibilities” and defined gender identity as “the feeling of congruence or incongruence in relation to the sex assigned at birth” and as a “function of the brain.” Researcher B's response delimits gender identity to “the interaction between very complex functional brain networks” and remains within a biologically deterministic framework. The implication is that researcher B does not consider biosocial frameworks as very relevant to the field, but rather than acknowledging or actively rejecting this alternative framework, the researcher blocks the dialogical space with their own theory.

Researcher C takes a synthesizing approach in which the SR/BP hypothesis is combined with the O/A hypothesis. This model considers “sex hormones and especially puberty” as “extremely important” for the development of gender identity and the concept of the self, but includes a dimension described as “identity development in general, so “how ok you are yourself with your body? how positive or negative you think about yourself.” Not just in terms of body image, but more generally.” This latter aspect could be interpreted as a possible opening to biosocial thinking, although it is presented in an additive rather than interactionist or dynamic manner.

#### Brain Sex Dimorphism and Brain Mosaicism

Here, I interrogated the stance of the interviewees regarding brain mosaicism as a conceptualization of brain differences between men and women, as well as the critiques of the dimorphic model.

Researcher B stated to “know the work that you mean” and moved on to reject it based on its lack of correspondence with their own data: “I don't agree with that kind of approach because it's not what I've seen.” However, in the next sentence, researcher B expresses a different stance without acknowledging the contradiction, reframing the brain mosaicist model as a political attitude: “I understand the feminist attitude and agree that dimorphic differences (two different forms) are observed only in the reproductive system and that the rest of the differences can be called sex effects.” Despite the affirmation of knowing this line of work, Researcher B misrepresents the brain mosaicist approach, which does not hold that the measured traits in the brain are “sex effects,” but dynamic interactions between multiple social and biological factors.

Researcher B goes on to emphasize the importance of the sex differences in relation to “morphology, physiology, behavior,” as well as “genetic expression,” “prevalence of psychiatric and neurodegenerative diseases,” “pharmacokinetics and pharmacodynamics,” and “neuroimmunology,” finishing with the rhetorical question “How do we explain all this from environmental factors and from a theory of patriarchy? Impossible.” Again, the researcher misrepresents the mosaicist model as denying or downplaying existing differences between people of different sexes/genders and frames it as a political theory.

Researcher C does not directly present their own stance on the question but instead reports a situation in which a proponent of the mosaicist model reached out to a supervisor of the researcher whose research is situated within a dimorphic model, in order to collaborate and test the mosaicist hypothesis with the data of the supervisor. Researcher C argues that “it would have been of value to collaborate on that part, I think it is very relevant,” acknowledging the value of the brain mosaicist approach.

#### Intersectionality and Categorization

I asked the interviewees to consider whether intersectional approaches should inform neuroscientific research and what difficulties this would entail. Researcher A engaged openly with the question, thinking about the relevance of the category “race.” Researcher A makes the appropriateness of an intersectional approach dependent on the research question and argues that it might be important to include the category “race” in the study of “gender identity” if a researcher wants to account for the fact “that stress or being in a minority position affects your brain.” When speculating about the possible ways in which race, gender identity and context of upbringing might interact with each other and affect brain development, Researcher A raises the problem of the feasibility of such a study: “there are too many millions of confounding factors which you cannot really control for.” In the response, Researcher A shows an understanding of intersectionality, engages with its implications and points to the limitations of neuroimaging studies for a complex understanding of gender identity development.

Researcher C, on the other hand, is unfamiliar with the concept but engages with it after asking me to explain it with an example. Researcher C proposes the use of covariants such as race or sexual orientation as a way to introduce an intersectional perspective. This misses the point of intersectionality as covariants follow an additional logic of the different factors and work toward the isolation of one “pure” factor, while the idea of intersectionality is precisely the entanglement of the different dimensions. Thinking about the interactions between the categories of gender identity and sexual orientation, Researcher C recalled unexpected findings where “cis lesbian groups” have values in brain measurements that “are even more male-typical than the trans males” and wonders “what's going on there? Did they use anabolics, for example?.” This train of thought reflects the difficulties in moving away from a paradigm of clear categories, as well as the tendency to focus on biological and quantifiable factors. However, beyond disciplinary and methodological barriers, the inaccurate understanding of intersectionality might also reflect my inability to make these points clear in the context of the interview.

#### Operationalization of Gender Identity in Cisgender Participants

I asked the interviewees to explain how the gender identity of cis participants was assessed in the studies that they were involved in.

Researcher A reported first that they were assumed but then became unsure and pointed to the principal investigator as the person who could answer my question.

Researcher B refused to answer and left the question out of the edited transcript altogether.

Researcher C recalls using a questionnaire with subscales for both cis and trans participants but adds “we never mentioned that, that's true.” The researcher explains how for cis participants “who have never had any identity issues, it's the most simple question to ask: are you a boy or a girl? They say 'yeah, of course, I am that'.” Since I was interested in the theoretical or conceptual challenge of understanding gender identity for cis and trans people, I pointed to the fact that “it can still mean different things when two cis people say “I am a boy” or “I am a woman.” What that means can still vary because they have different ideas of what that means.” Researcher C agreed with the importance “from a methodological point of view” to “characterize your sample in a more detailed way,” but immediately linked this to controlling “that none of your cisgender people struggles with identity issues.” The response shows again that the conceptualization of gender identity in the BSGI is constructed around the distinction of trans/incongruent and cis/congruent, erasing the complexity of the category. The rationale offered is a pragmatic one: “the simple distinction is to include someone with a diagnosis and those who not.”

#### Gender Diversity and Non-binary Identities

The interviewees were asked about the implications of the acknowledgment of non-binary gender identities in the DSM-5 for the field. The question aimed to challenge the assumption of bipolar and dichotomous gender identities that dominated the BSGI.

Researcher A explains the novelty of non-binary identities as a result that “few people told us” in the beginning, “even if they of course existed.” Researcher A explains the exclusion of non-binary participants because in “this type of research you need to have, in quotation marks, “clean” group as possible.” The acknowledgment of non-binary identities leads researcher A to a profound interrogation of the meaning of gender identity in both trans and cis populations “(.) if you ask 10 cisgender women how can you describe your female gender identity? you get different explanations. That's the main problem with gender identity, that it is so subjective for each individual.”

For researcher B, non-binary identities are contained within gender incongruence as a “minority that is not binary, present incongruence with the assigned sex or feel that they belong to another gender, or experience changes over time with respect to gender identity, or feel that they do not belong to any gender.” This framing of non-binary identities leave the binary model of two genders and distinction congruent/incongruent as structuring notions largely unchallenged.

Researcher C welcomes changes in terminology as less stigmatizing for trans people but expresses difficulties grasping non-binary identities. Researcher C states to not really “understand what it is to be gender non-binary,” unlike binary trans people, who “request testosterone treatment then and surgical changes (…) in order to get my body the way I feel I am.” The medicalized trajectory of a binary sex change is a real phenomenon to Researcher C, but not non-binary identities. Researcher C wonders “how real that phenomenon is? Is that really (…) from people who experience actually this feeling and who only now dare to share that, or is it more some kind of trend that allows you to get the autonomy of defining yourself as whatever you like because it is possible?.” The influence of cultural context on the articulation and expression of gender identities (“trend”) is used to challenge and potentially dismiss non-binary identities as a form of fiction, while unexplored and unaccounted in relation to binary masculine or feminine identities and their relative social privileges. This view shows a bias toward a binary model, but also represents an awareness of the deep implications of non-binary identities for the BSGI and the challenges it poses to current models.

#### Understanding of Transgender Identities

My question about the changing criteria that define gender incongruence in the DSM-5 was a way to engage the interviewees in a conversation about transgender identity and gender identity overall as shaped by both biological factors and socio-cultural factors, moving away from the biologically deterministic models of the BSGI.

Researcher A demonstrated a complex understanding of transgender identity. Researcher A takes into account self-determination as a first component: “we ask the patient “what do you call your gender identity?”.” The second component is forms of distress stemming from the self, and the third is forms of distress stemming from the social environment: “in what way does that gender identity mismatch or distress you when you look at yourself or think of your own body? (…) how much does the distress that surrounding sees you, misgenders you, or belonging to that gender role?.” Another aspect is a critique of othering and of the distinction between cis and trans: “we should stop seeing trans people as exotic or special, where there are more things in common.” Researcher A situates gender identity and perceptions of the body in relation to normative ideas of masculinity and femininity: “the way you think about your body is reflected from what society norms. Like, old female bodies are not nice, but 25-year-old females in a cis heteronormative world are, so. It's probably impossible to think, to separate them and to even know. Am I unhappy of my breast size due to that I'm really unhappy about them or that there are society norms for breast size?.”

Researcher C, has an understanding of transgender identities tied to diagnostic categories and biological factors. I urged the researcher to take into account the historical and cultural dimension and think about “how, before there was something called trans, did people who now would be understood as such, live, and what ways of understanding themselves did they have?.” In their reaction, the researcher first focuses on the role of technology and techniques as means to express gender identity, such as “medical possibilities” and “the internet,” where “you can photoshop yourself until it fits the identity you have actually.” This follows a logic of “true” and “fake” identities and the sense of a prior gender identity as stemming from the self. But researcher C then elaborated on the development of cis or transgender identities in a triad of “sexual maturation” and “interest in the other, usually in the opposite sex,” “reorientation with social changes from family to peers” and “thinking about yourself, who am I, not only in terms of boy or girl, but also in terms of who am I in this world.” This understanding of transgender identities, while still focused on the cis and trans distinction is much more complex than the definitions found in the BSGI and shows many possibilities for introducing contextual factors as constitutive of gender identity development.

## On Epistemic Injustice in Brain Studies of (Trans)Gender Identity

In this section, I finally address the central question of my paper “Which forms of epistemic injustice can be identified in the conceptualization of gender identity in the brain studies of (trans)gender identity?.” Before presenting my conclusions, certain remarks on the validity of this research are due. My analysis is based on my open alignment with biosocial, developmental, mosaicist, contextualist and depathologizing approaches, I am not a neutral observer but a situated agent. Therefore, the whole project is founded on acceptance of feminist and social epistemologies of science. Regarding my analysis of the conceptualization of gender identity in BSGI, the results are limited to the sample and can't be automatically extrapolated to represent similar BSGI. Regarding the expert interviews, it needs to be taken into account that these types of interviews are not meant to provide results to be generalized. My assessment of the interviewees' epistemic attitudes only holds true, in a strict sense, in the context of the dialogue which unfolded in the interview and cannot be assumed to characterize past or future positions of the interviewees. Regarding the assessment of epistemic attitudes, the categories of analysis offer a margin for interpretation. Perceptions of epistemic behaviors might vary between different analysts, as well as judgments of relevance of different dimensions. Despite these restrictions, the findings of my analysis are consistent and relevant enough to open up a critical discussion of the conceptualization practices identified in the studies.

### Testimonial Injustice in Published Brain Studies of (Trans)Gender Identity

In the early published studies biosocial, developmental, mosaicist, contextualist and depathologizing approaches were completely ignored. Taking into account the direct relevance of these approaches as well as the responsibility of researchers to engage with the current state of knowledge on the topic of research, I argue that the early published papers represent a form of active silencing or blocking of these lines of work. The exclusion of this knowledge is connected to the conceptual problems identified in the studies, namely internal conceptual inconsistency, hermeneutical misconception and the upholding of questionable paradigms. The epistemic injustice involved in the exclusion of counter-hegemonic positions represents at the same time a failure of the epistemic system of empirical scientific work.

For the recent studies, I want to acknowledge that while biosocial researchers were not explicitly acknowledged, the CD and SR/BP hypotheses mention environmental and experiential factors. However, this is not reflected in changes in research design nor in an adequate theoretical discussion, which is why I argue that the testimonial exclusion of scientists working on biosocial approaches of sex/gender is perpetuated in the more recent studies, but acknowledge that the theoretical opening holds the possibility of a future correction. Also, while the criteria for gender dysphoria in the DSM-5 are incorporated, there is no engagement with the conceptual implications of the changes. Instead, the CD hypothesis relies on Blanchard's typology of trans, and the SR/BP hypothesis erases gender as a dimension.

The changes introduced in the recent studies show that the epistemic system of the BSGI has a selective sensitivity. It responds to dissonance between predicted findings and observed findings, giving impetus to the search for new theoretical references and modification of the O/A framework. Despite these developments, the problems of conceptual inconsistency, hermeneutical fallacy and questionable paradigms persist.

But can the epistemic agents involved in the BSGI be said to benefit from the exclusion of biosocial, developmental, mosaicist, contextualist and depathologizing approaches? To answer this question, it is helpful to consider Latour's account of the establishment of scientific facts. He shows the facts are established as such through the uptake and use by other researchers to ground further claims. In this sense, the testimonial silencing of alternative approaches has two effects. First, it prevents a challenging of the claims upon which the BSGI are built, strengthening the research's value in terms of credibility. This results in relative career advancement and greater access to grants, for example. Second, through silencing, the BSGI actively work toward an exclusion of the positions of the alternative approaches as scientifically relevant. These effects combined make it possible that the epistemic agents involved in the BSGI suffer no loss of epistemic status or credibility despite the deficiencies of the knowledge produced. Further, the epistemic agents of the BSGI can generate more studies and results through not engaging with complex conceptual questions, which would be a time-intense form of work with less revenue than the production of empirical data. From this examination, I conclude that the testimonial silencing and lack of sensitivity toward biosocial, developmental, mosaicist, contextualist, and depathologizing approaches in the published studies of the BSGI represent a case of epistemic injustice that needs to be addressed.

### Testimonial Injustice in the Research Process of Brain Studies of (Trans)Gender Identity

The first insight from the interviews on the question of testimonial injustice against biosocial, developmental, mosaicist, contextualist, and depathologizing approaches in the BSGI is the visibility of different positions of the researchers involved in the BSGI. The epistemic attitudes toward the alternative approaches ranged from acceptance and familiarity, favorable assessments and openness, to resistance and blocking. Further, the interviews also showed the role of the hierarchical organization of research teams in the suppression of dissent and alternative approaches. This is enabled by concentration of the decision-making power in the role of the principal investigator, who is also the receiver of funds and the owner of research data. The fact that epistemic agents directly involved in the BSGI, such as researcher A, are familiar and favorable to counter-hegemonic approaches, or open to engage with them, such as researcher C, suggests that one mechanism of the epistemic injustice is through epistemic devaluation of dissenting voices within research teams, especially the ones of subordinated researchers.

The second insight from the interviews was the identification of specific instances of harmful testimonial practices against proponents of counter-hegemonic approaches, such as the refusal to collaborate reported by researcher C or their active devaluation as unscientific by researcher B. Both instances are enactments of willful ignorance, of not wanting to know. In the first case, the knowledge that could be gained through the collaboration is blocked. In the second case, there is a need to suppress certain knowledge in order to maintain an epistemic situation, despite the dissonance embedded in this suppression.

A third insight was that besides targeted forms of exclusion, there are more diffuse forms of testimonial exclusion at work in the BSGI. One instance is the ignorance of social theories of sex and gender exemplified by researcher B and C, showing the insensitivity of the epistemic system to the exclusion of whole disciplines. A second instance is the devaluation of the claims of epistemic agents that were perceived as motivated by political interests, such as feminist scientists, “militant” or “activist” researchers. Noting that only counter-hegemonic positions challenging the status quo of society are perceived as political, while positions resisting change are perceived as neutral and capable of objectivity, this exclusion indicates a different kind of failure of the epistemic system of the BSGI. It is an epistemic system that is not able to reflect and integrate into its knowledge production process the positionality of its researchers in relation to their topic of research. It fails to account for the ways in which the situatedness of the researchers shapes perceptions, categories, hypothesis or interpretations. This critique has been also raised from within the field of neuroscience of gender identity (see Walsh, 2015).

I argue that the active and targeted exclusions of counter-hegemonic approaches and their epistemic agents is enabled and promoted by systemic factors related to the organization of scientific work such as the projectification of science, which tends “to privilege already codified over novel, uncertain knowledge; theory and application of methodology over building upon it; hypothesis testing over creation or, in short, “normal science” over revolutionary, risky or unorthodox science” (Torka, 2018, p. 61). On this basis, strategic epistemic practices can be suggested toward developing a higher sensitivity toward the diffuse and targeted exclusion of alternative and counter-hegemonic approaches: individual openness, transparency of internal disagreements and multiplicity of interpretations within research teams, the promotion of exchange across disciplines, building inter- and transdisciplinary networks and collaborations centered on a common question, open data initiatives, and the reassessment of funding and review criteria in order to promote theoretical innovation and sensitivity to the exclusion of marginalized or counter-hegemonic approaches.

### Underlying Hermeneutical Deficiencies in the Brain Studies of (Trans)Gender Identity

One hermeneutical deficiency of the BSGI stems from the suppression of critical epistemologies in biology and natural sciences. These critical epistemologies have been informed by an acknowledgment of the social embeddedness of research and knowledge, as has been shown by works from history and sociology of science. It is only through the suppression of these works that the internal/external division between science and society can be upheld. Critical epistemologies also challenge the notion of “nature” as that which is really true, and as opposed to phenomena that are seen as socially constituted. In order to move toward a more epistemically just situation it is important to establish an understanding of science that is able to acknowledge the situatedness of research and the hybrid social and biological constitution of phenomena.

A second hermeneutical deficiency results from the historic epistemic oppression of trans people as epistemic agents. The current regime of legal and diagnostic procedures is built on a denial of credibility of trans people regarding their own gender identities. Despite the move toward depathologization of medical and clinical vocabulary, the epistemic oppression of trans people persists in the dependence on medical experts. The BSGI are not only embedded in this epistemic situation, they also enact this same devaluation of trans people's credibility. This is exemplified in the different assessment procedures to determine the gender identity of trans and cis participants, as well as the difficulties to acknowledge non-binary identities as a real phenomenon. The historical suppression of trans and gender diverse people's statements regarding their gender identity has created a hermeneutical system that lacks the resources to make sense of the existence of trans and gender diverse people, and that is inadequate to understand gender identity in all its expressions.

I argue that the instances of testimonial injustice against the epistemic agents of alternative approaches in the BSGI are secondary to the historic epistemic oppression of trans and gender variant people, and to the suppression of critical epistemologies. These suppressions lead to hermeneutical deficiencies that cause the BSGI's epistemic system in its current practices and structures to generate deficient knowledge about gender identities. In order to move toward an epistemically just situation, changes in science education are necessary, such as the introduction of pluralist and critical epistemologies. Further, the administratively inscribed epistemic devaluation of trans people in procedures for legal name and sex change and access to trans healthcare needs to be dismantled. Only then, by ensuring the autonomy of trans people from medical and scientific authorities in their access to fundamental rights and involving them in the research process, can a situation be generated in which trans people regain epistemic agency and trust.

## Data Availability Statement

The raw data supporting the conclusions of this article will be made available by the author, without undue reservation.

## Ethics Statement

Ethical review and approval was not required for the study on human participants in accordance with the local legislation and institutional requirements. The patients/participants provided their written informed consent to participate in this study. Written informed consent was obtained from the individual(s) for the publication of any potentially identifiable images or data included in this article.

## Author Contributions

The author confirms being the sole contributor of this work and has approved it for publication.

## Conflict of Interest

The author declares that the research was conducted in the absence of any commercial or financial relationships that could be construed as a potential conflict of interest.

## Footnotes

^1^The listed transdisciplinary contributions are not exhaustive, they can be expanded with further critical works from situated Neuroscience and psychological studies, which were not taken into account in my research.

^2^“female” = XX, ovaries, uterus, fallopian tubes, vagina, labia minora and majora, clitoris, and “male” = XY, testes, prostate, seminal vesicles, scrotum, penis.

^3^In order to establish a dialogue between different disciplines and approaches, I chose a pragmatic approach to the use of terminology. The terms “transgender”/“trans” and “cisgender”/“cis” are extracted from the field, following the vocabulary of recent neuroscientific papers (see Burke et al., 2017; Nota et al., 2017; Manzouri and Savic, 2018 for example) that seemed the most compatible with terms as used in gender and trans studies. The same applies to the use of “gender identity”, taken from the field. The downside is that through this pragmatic approach, limitations, and problems bound to these terms are reproduced.

^4^I am limiting the epistemic agents in my analysis to those who actively participate in the production of knowledge within the delimited epistemic situation because of my focus on the question of responsibility and accountability for unjust epistemic practices. This excludes the cis and trans participants from my consideration.

^5^The Supplementary Materials include an extended report on the selection procedure and a full list of the analyzed terms.

^6^I excluded Rametti et al. (2011b) from the quantitative assessment because of the parallels in use of the terms to Rametti et al. (2011a).

^7^See full interview script in the Supplementary Material.

^8^The concept of social field follows Bourdieu's definition of social field as “a field of forces, whose necessity is imposed on agents who are engaged in it, and as a field of struggles within which agents confront each other, with differentiated means and ends according to their position in the structure of the field of forces, thus contributing to conserving or transforming its structure” (Bourdieu, 1998, p. 32).
