# Supplementary material for: Epistemic Injustice in Brain Studies of (Trans)Gender Identity
Source: Front Sociol. 2021 Mar 26;6:608328. doi: 10.3389/fsoc.2021.608328 (PMC8022811; doi:10.3389/fsoc.2021.608328)
Supplement: Supplementary file 1 [file Data_Sheet_1.doc]

Supplementary Material

Extended report on the selection process of brain studies of (trans)gender identity:

Using PubMed, Google Scholar and ScienceDirect, I searched for publications including the terms “transgender brain, transsexual brain”, “transsexualism and brain”, published between 2000[[1]](#footnote-2) and 2018. After deleting double entries and unrelated papers, I found 7 review papers, 87 original studies and 17 theoretical papers. I quantified a total of 337 researchers involved. From 337, 16 researchers had published 7 or more papers, 25 had published between 4 and 6 papers, and 71 had published 2 to 3 papers. I then analyzed the co-authorship of the 41 researchers with 4 or more publications on the topic, leading to the identification of four research hubs in Europe.

I first read the reviews and theoretical papers. These allowed me to identify which papers were considered to be key studies of the field. I added missing studies to my database. I then reviewed the studies for their own definitions of research question, theoretical framework, hypothesis and interpretation of the findings. I narrowed the sample to studies focusing on gender identity as their explicit object of research. I excluded neuroimaging studies with trans participants looking into the effects of hormone replacement therapy on brain structure or function (see for example Burke et al., 2018), as well as studies with research object that were not explicitly gender identity[[2]](#footnote-3), such as ostracism (for example Mueller et al., 2018) or reaction to stimulation of body parts (see for example Case et al., 2017).

**Full list of analyzed terms in the published brain studies of (trans)gender identity:**

In order to account for the conceptualization of gender identity in the published BSGI, I included the following:

1. Explicit definitions of “sex”, “gender”, “gender identity”, “sexuality” and “trans / gender dysphoria / transgender / transexual”
2. Use of “sex”, “gender”, “trans / gender dysphoria / transgender / transexual”, “women / female / feminine”, “men / male / masculine”, and terms to define sexual orientation such as “homosexual”, “bisexual” and “heterosexual”.
3. Expressions of sexual differentiation of the brain: “feminization”, “masculinization”, “sex dimorphic”, “sex differentiated”, “sex a/typical”, comparisons of measurements between “men”, “women”, “FtM” and “MtF”, and comparisons of measurements between “biological sex” and “gender identity”.

Full interview script

1. Introduction and contextual information
   1. Can you explain when and how you decided to work on brain studies of transgender identity?
   2. What was your interest or motivation on the topic?
   3. Could you briefly explain your role in the transgender brain studies that you participated in?
   4. Apart from the interest you mentioned, did other aspects play a role in your decision to work on the neuroscience of transgender identity, such as personal or political reasons, career perspectives, or possibilities to collaborate with other researchers?
2. Self-positioning and establishing a common ground
   1. In the studies, the findings don’t support the original hypothesis of brain sex reversal. Based on the findings, Guillamón and colleagues have formulated the cortical development hypothesis. An alternative hypothesis has been proposed by Savic and her team, which focuses on self-referential thinking and body perception. Regarding these developments in the field, I am interested in two issues: first, the construction of gender categories beyond the male-female dichotomy and second, the incorporation of biosocial theoretical frameworks. In a general sense, what is the relevance of these two aspects for the neuroscientific study of gender identity and gender incongruence?
3. Attitudes to alternative conceptualizations in relation to the transgender brain studies
   1. In the studies the brains of trans people have been described as intersex or a mosaic of male and female traits. Feminist neuroscientists however, emphasize that at an individual level, apart from some specific sex-dimorphic areas, brains of men and women can also be understood as intersex, that is, as a mixture of statistically "male" and "female" patterns. They question the notion of brain sex dimorphism. Was the appropriateness of working with the two constructs of “male brain” and “female brain” discussed in the studies that you participated in?
      1. What relevance holds the principle of brain mosaicism for the neuroscientific research of gender identity and gender incongruence?
      2. Considering that the definition of the trans brains is routinely done in relation to male-type and female-type brains, what do you think the implications of leaving a brain sex dimorphism behind are for thinking about cis and trans gender identities?
   2. Wendy Wood and Alice Eagly (2012) have proposed a biosocial concept of gender role socialization. In this model, socialization pressures act on individual and sex-based different biological predispositions. How relevant do you think knowledge on socialization trajectories of cis and trans men and women is for the neuroscientific study of gender identity?
      1. In which way was this body of work taken into account in the transgender brain studies that you have worked in?
      2. Anne Fausto-Sterling and colleagues (2011a, b) have proposed a Dynamic System Theory-based biosocial model on the emergence of sex-differences in children. This approach looks at subsystems affecting bodily differentiation, behavioral differentiation and gendered knowledge in their development. How relevant do you think approaches attempting to account for the dynamic interactions between social and biological subsystems over time are for the neuroscientific study of gender identity and gender incongruence?
      3. What problems and benefits at a theoretical, methodological or practical level do you see in this or similar dynamic biosocial approaches?
   3. In addition to the mentioned approaches by social psychologists and feminist biologists, feminist neuroscientists question interpretations of brain differences between men and women as determined by the organizational effects of prenatal hormones. They draw on the principle of brain plasticity and on empirical findings showing environmental factors modifying, neutralizing or reversing brain sex differences. Do you think neuroimaging studies on gender identity should move away from a hardwiring paradigm towards incorporating the contingency and dynamism of brain sex differences?
   4. In the transgender brain studies, sexual orientation is defined as a biological factor and operationalized based on the genitals at birth. For example, a transwoman sexually attracted to cismen in categorized as homosexual. This definition negates the experience of sexual orientation of trans people, as well as that of their partners. Was this contradiction between the social experience of sexual orientation and the biological concept of sexual orientation a topic of discussion?
      1. Within intersectional approaches, gender is defined as a multi-dimensional construct that integrates sexual orientation among other relevant social categories such as class or race. Do you think that intersectional studies should inform neuroscientific research on gender identity and sexual orientation?
   5. The diagnostic criteria for gender dysphoria and gender incongruence in the DSM-5 and ICD-11 incorporate a series of conceptual changes. The criteria A in the DSM-5 reads: “A marked incongruence between one’s experienced or expressed gender and assigned gender ...”. This formulation reflects a shift away from a binary gender logic of male/female, replacing formulation such as “cross-gender” or “opposite sex”. According to the members of the committee, this was done to acknowledge the variety of self-definitions of trans people, as well as the fact that many trans people do not wish to undergo a complete hormonal and surgical transition. In the transgender brain studies, there is only male and female identities for trans people. Was this decision discussed at a theoretical or methodological level in the studies that you were involved?
      1. What implications do you think this acknowledgement of a diversity of identities in transgender people has for the neuroscientific study of gender identity in general?
   6. The next question looks at how this reconceptualization of gender could be put into practice in research. In a study on gender identity from 2013 feminist neuroscientist Daphna Joel and colleagues developed the Multi-Gender Identity Questionnaire to measure gender identity in non-trans individuals. The questionnaire assesses for all participants items such as feeling like a woman, like a man, like both or like neither, contentment with affirmed gender, wish to be the ‘other’ gender, contentment with one’s sexed body, or compliance with gender norms in clothing and language. What benefits and problems do you see in this operationalization model for neuroscientific research on gender identity and gender incongruence?
   7. The main theoretical model of the transgender brain studies proposes that the determination of genital phenotype and brain phenotype are independent of each other. Because of this, I would assume that it is necessary to assess the gender identity of all participants in order to make valid inferences. But in my analysis of the transgender brain studies, I could find no information about whether or how gender identity was assessed in the cis participants. Could you elaborate on how the gender identity of the cis participants was determined in the studies in which you participated?
      1. Was this discussed as a theoretical and methodological problem in the studies that you were involved?
      2. The findings of the mentioned study by Daphna Joel and colleagues, which is informed by approaches from Gender Studies, show that gender identity is not a unitary feature for cis men and women. Over 30% of cismen and ciswomen felt to some extent as the ‘other’ gender, as two genders and/or as neither gender. Considering this, should the gender identity of the cis participants in neuroscientific research on gender identity and gender incongruence be assessed?
   8. The next set of questions deals with how the diagnostic definition of gender dysphoria and gender incongruence was used in the studies. In the debates preceding the release on the ICD-11 and DSM-5, it is clear that the definition and criteria of gender dysphoria and incongruence are guided by therapeutic utility, considerations of access to health and legal services, and the reduction of stigma. It is not intended as a gender identity assessment tool, but it is used as such in the transgender brain studies. Was the use of the diagnosis of gender incongruence as assessment of gender identity a topic of discussion in the studies that you participated in? Were other options for assessing the gender identity of trans participants discussed?
   9. The definition of gender dysphoria in DSM-5 conceptualizes incongruence as the discrepancy between experienced or expressed gender and assigned gender. This implies that the incongruence emerges from the interaction of the self and the socio-cultural gendered norms and expectations that prevail in a society. However, in the transgender brain studies working within the brain organization/activation theory, the incongruence is conceptualized as an individual biological trait. Was this a topic of discussion in the transgender brain studies that you participated in?
      1. Do you see as a legitimate option to ignore the socio-cultural dimension of gender incongruence in the neuroscientific research even if it is considered central in the diagnostic definition?
      2. Do you think that neuroscientific research on gender incongruence would benefit from incorporating the sociocultural dimension of gender in the design of experiments and interpretation of findings?
   10. In Savic’s line of research, they look at neural networks involved in self-referential thinking and body perception. These neurological models don’t include gender as a category of analysis. The incongruence is defined in these studies as an incongruence between one’s sense of self and one’s body, leaving gender out. But if the incongruence of trans people is defined in the DSM-5 and ICD-11 as a gendered process, what are the theoretical benefits of using a conceptual framework that can’t account for gender differences?
       1. To which extent does this bracketing out of gender in the definition of incongruence distort the phenomenon under study?
       2. Is it possible to make valid theoretical inferences on gender incongruence from a sex and gender-neutral framework?

1. The three studies that first defined the TBS field were published in 1995 (Zhou, Hofman, Gooren & Swaab), 2000 (Kruijver et al.), and 2008 (Garcia-Falgueras & Swaab). 2000 was therefore considered a safe starting point for a first mapping. Earlier relevant publications would be identified through analysis of the cited papers. [↑](#footnote-ref-2)
2. This does not imply that these studies are not relevant for an understanding of gender identity in cis and trans people. [↑](#footnote-ref-3)
